# Linking abnormal neural activity patterns to motor deficits

**DOI:** 10.7554/eLife.100833

**Published:** 2024-07-24

**Authors:** David J Herzfeld

**Affiliations:** 1 Department of Neurobiology, Duke University School of Medicine Durham United States

**Keywords:** cerebellum, ataxia, dystonia, tremor, classifier, optogenetics, stimulation, Mouse

## Abstract

Abnormal activity in the cerebellar nuclei can be used to predict motor symptoms and induce them experimentally, pointing to potential therapeutic strategies.

**Related research article** van der Heijden ME, Brown AM, Kizek DJ, Sillitoe RV. 2024. Cerebellar nuclei cells produce distinct pathogenic spike signatures in mouse models of ataxia, dystonia, and tremor. *eLife* **12**:RP91483. doi: 10.7554/eLife.91483.

The brain is made up of a dense web of interconnected brain regions. This means that when neurological dysfunction occurs in one area, errant signals can spread to connected regions. While the spread of abnormal activity in the brain poses challenges for researchers developing therapeutic interventions, it also presents opportunities. It might be possible to detect abnormal brain activity and correct it without needing to pinpoint the exact origin of the dysfunction.

Studying movement disorders is an ideal medium for understanding the effects of neurological dysfunction on brain circuits because animal models that mimic the motor symptoms of patients can be studied in the laboratory. The cerebellum is one of many areas crucial for movement control and presents a unique opportunity to understand how neural circuits are linked to motor disorders (Holmes, 1917). Information from much of the brain is sent to the cerebellum and processed by expansive neural circuitry in the cerebellar cortex, the folded surface of the cerebellum. The processed inputs are then funneled through an information bottleneck at the synapse between Purkinje cells, which are the sole output of the cerebellar cortex, and their targets in discrete clusters of neurons in the cerebellar nuclei (a set of relay stations located deep in the cerebellum). From there, the information processed by the cerebellum is broadcast back to the rest of the brain (Bostan et al., 2013). The large number of inputs to the cerebellar cortex and the compression of information at the cerebellar nuclei provide an ideal target for understanding how dysfunction in one area can affect connected regions.

Now, in eLife, Meike van der Heijden, Amanda Brown, Dominic Kizek and Roy Sillitoe report that abnormal patterns of activity from a single set of neurons can be used to predict motor symptoms associated with multiple neurological disorders (van der Heijden et al., 2024). Taking advantage of the information bottleneck at the cerebellar nuclei, the team – who are based at Baylor College of Medicine and Texas Children’s Hospital – measured the firing patterns of these neurons in mouse models of movement disorders. The recordings were then used to devise a relatively simple machine-learning classifier, which examines the firing patterns and automatically sorts them into different categories based on their characteristics.

The classifier identified unique firing patterns associated with different motor symptoms and then successfully predicted motor symptoms across multiple mouse models of the same neurological disorder (Figure 1A). This predictive ability was true for motor symptoms often associated with cerebellar disorders, such as ataxia (loss of coordination), as well as those often associated with dysfunction of other brain regions, for example, dystonia (uncontrolled muscle spasms).

**Figure 1.**
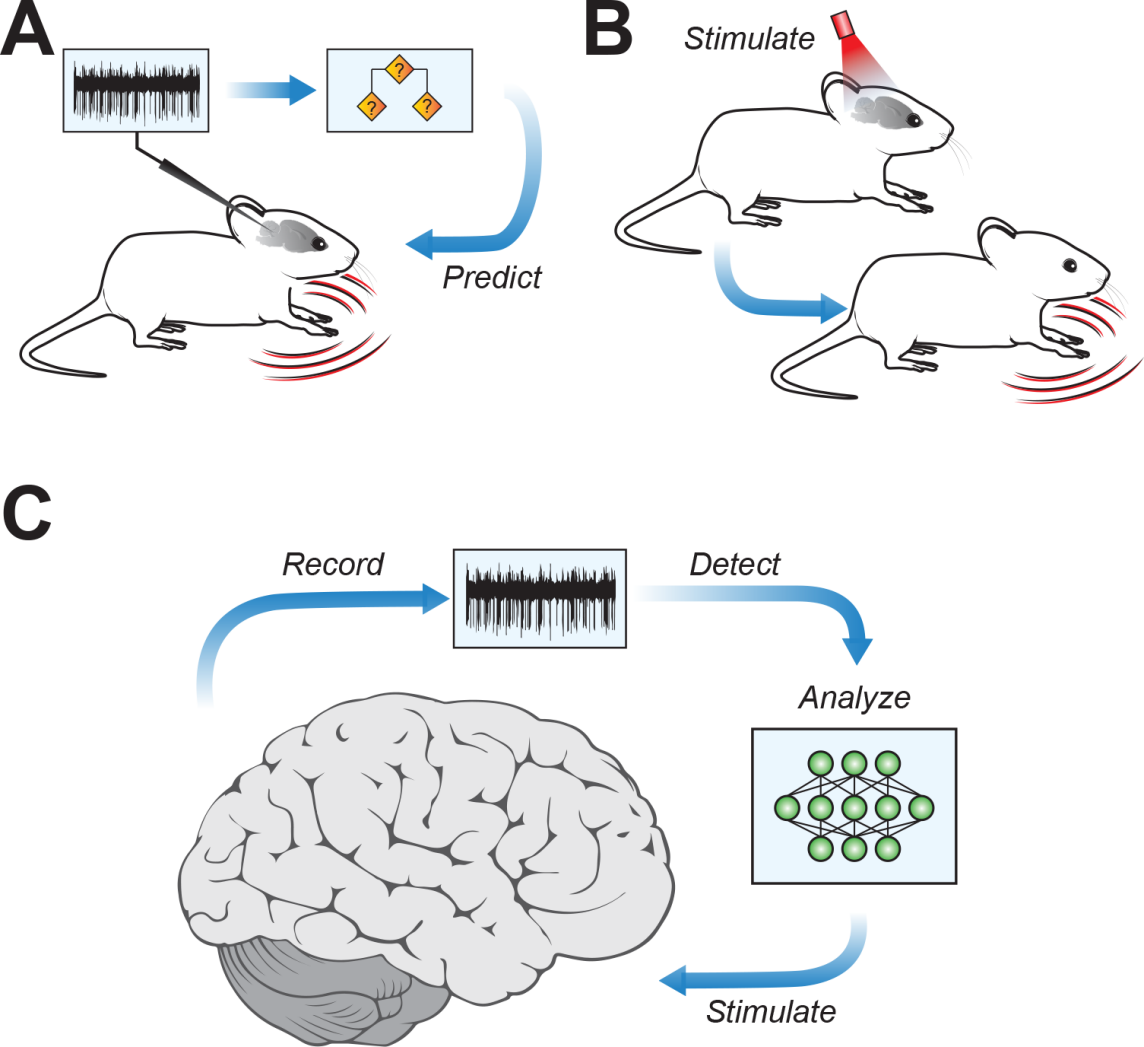
Connecting cerebellar nuclei activity to motor symptoms. (**A**) Using the recorded firing activity patterns of cerebellar nucleus neurons, a classifier can predict motor deficits in mouse models of motor disease. (**B**) Inducing abnormal activity in cerebellar nucleus neurons (using, for example, optogenetic systems) with specific stimulation parameters can cause motor symptoms in healthy mice, mirroring those in motor disease models. (**C**) Conceptual approach for closed-loop analysis and brain stimulation, where recordings of neural firing patterns are used to detect aberrant activity and to identify stimulation patterns targeting the cerebellar nuclei to potentially alleviate symptoms of neurophysiological diseases.

The team then went a crucial step further. If unique firing patterns in the cerebellar nuclei can predict motor symptoms, can inducing these patterns in the brains of healthy mice cause motor symptoms even in the absence of neurological disorders? Using an optogenetic system to modify firing activity in cerebellar neurons, van der Heijden et al. were able to induce motor deficits in mice that were reminiscent of motor disorders (Figure 1B).

The findings of van der Heijden et al. provide a major stepping stone for understanding the cerebellum’s role in motor disorders, and they also have broad implications for targeted therapies to reduce motor symptoms in patients. If driving aberrant activity in cerebellar nuclei neurons produces motor symptoms, then the opposite might also be true – strategically chosen patterns of stimulation might be able to restore healthy firing patterns and reduce motor deficits (Figure 1C).

It is possible to envision a device that includes a sophisticated classifier – perhaps leveraging advances in deep-learning techniques – capable of pinpointing precise patterns of aberrant activity in the cerebellar nuclei. This device could then identify the targeted stimulation parameters needed to push the aberrant activity back towards normal, a technique termed “closed-loop” brain stimulation (Chandrabhatla et al., 2023).

With monitoring of neural activity in carefully chosen sites and strategic selection of stimulation parameters, closed-loop brain stimulation could even be therapeutic for neurological disorders without motor dysfunction. It has only recently been appreciated that the cerebellum is also densely interconnected with regions of the brain traditionally thought of as ‘non-motor’ (Strick et al., 2009). In addition, it is now known that cerebellar damage often results in deficits in higher-order behavioral functions, such as difficulty with language and visuo-spatial cognition (Schmahmann, 2019). Therefore, targeted stimulation of the cerebellar nuclei might also alleviate some non-motor symptoms of neurological disorders. While significant research remains, the work of van der Heijden et al. highlights a bright future for cerebellar nuclei stimulation as a therapeutic target for a range of brain disorders.
